# Supplementary material for: Protective role of fructokinase blockade in the pathogenesis of acute kidney injury in mice
Source: Nat Commun. 2017 Feb 13;8:14181. doi: 10.1038/ncomms14181 (PMC5316807; doi:10.1038/ncomms14181)
Supplement: Supplementary Information — Supplementary Figures. [file ncomms14181-s1.pdf]

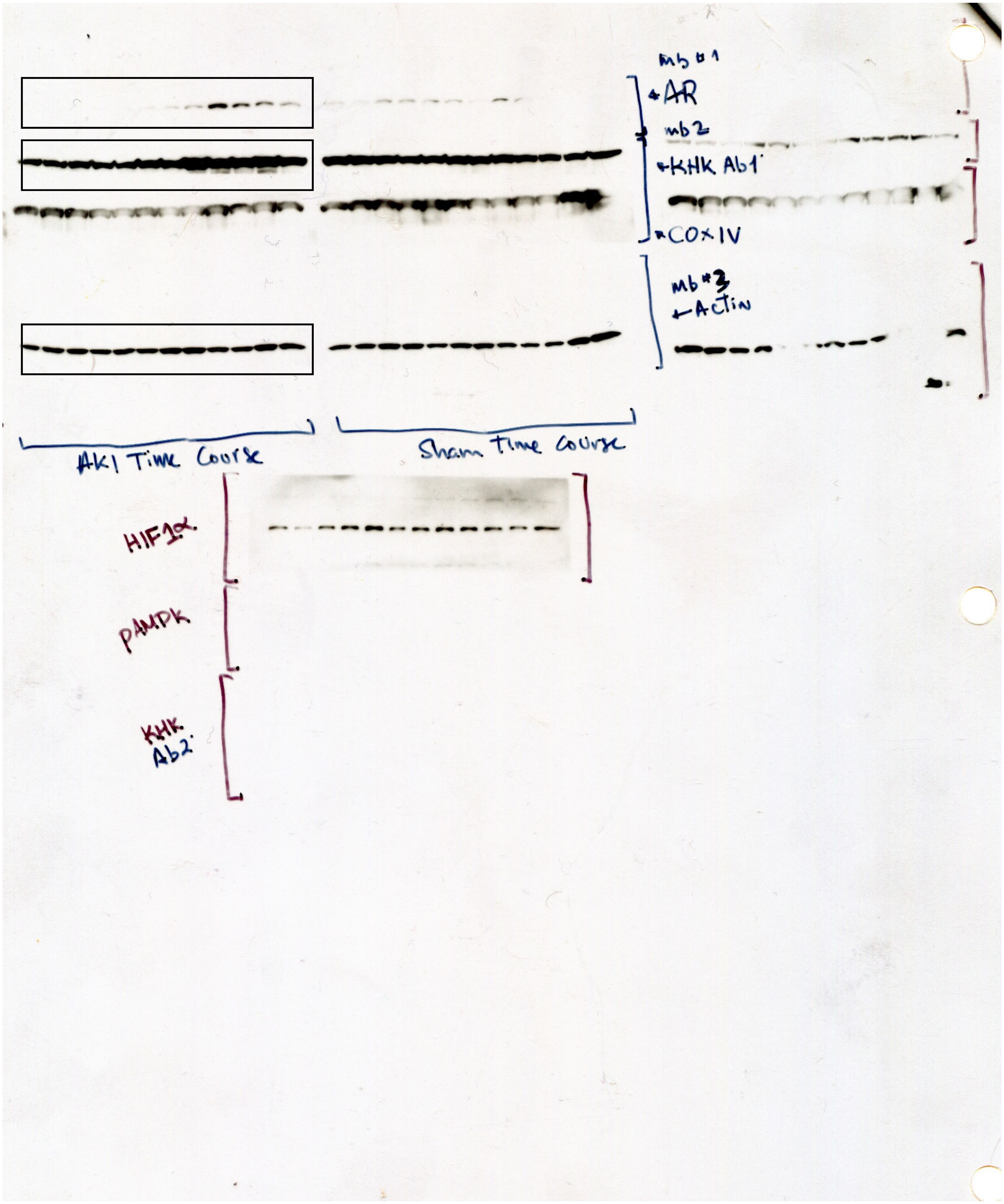

Supplementary figure 1. Whole film for western blot in figure 1

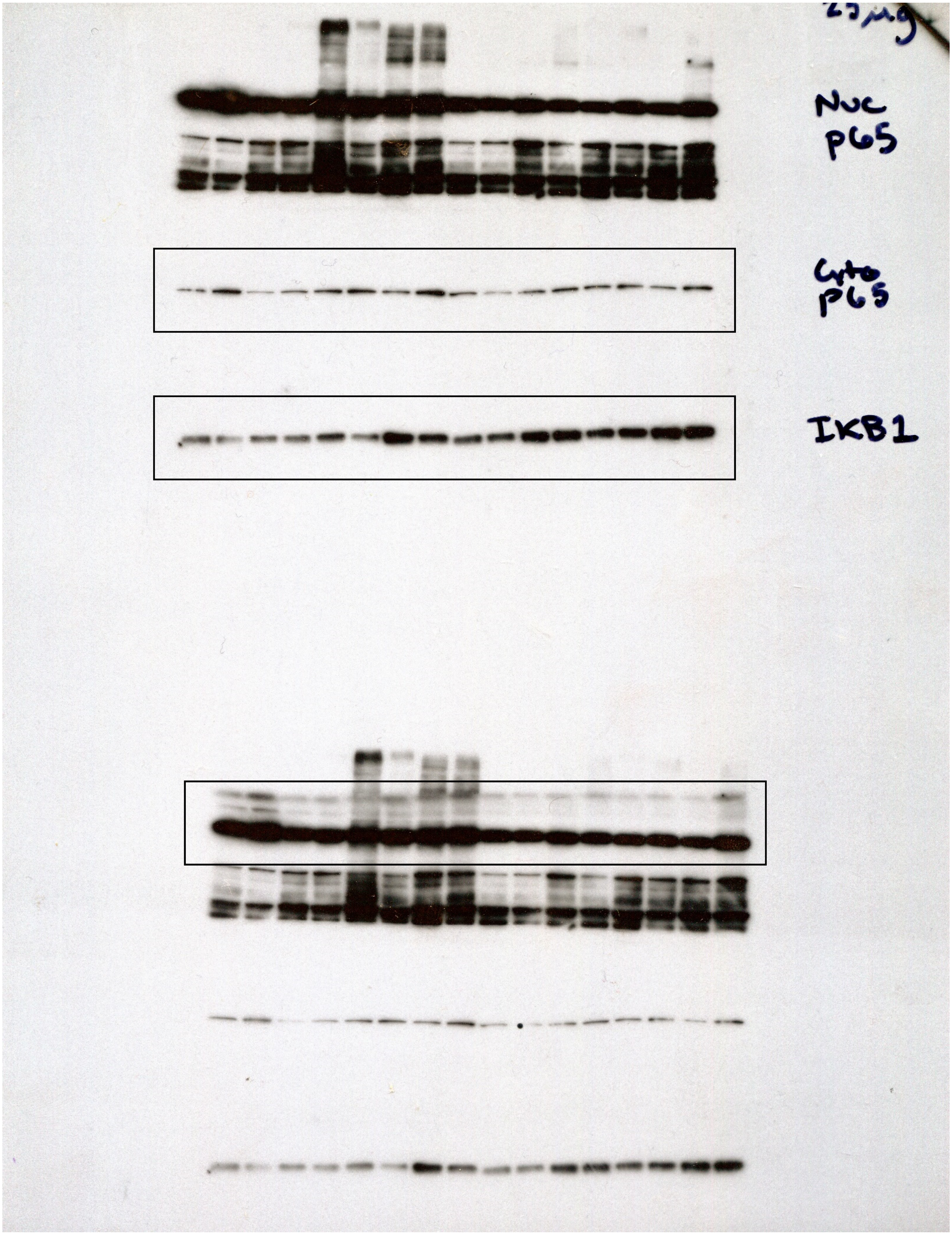

Supplementary figure 2. Whole film for western blot in figure 7
